# Provenance-Specific Height–Diameter Modeling for Chinese Fir: A Clustered Mixed-Effects Approach

**DOI:** 10.3390/biology14091301

**Published:** 2025-09-20

**Authors:** Xiangrong Wu, Yuhan Wang, Yanjuan Lyu, Wanrong Chen, Ming Li, Shuaichao Sun

**Affiliations:** 1 College of Forestry, Fujian Agriculture and Forestry University, Fuzhou 350002, China; 2 Chinese Fir Engineering Research Center of National Forestry and Grassland Administration, Fuzhou 350002, China

**Keywords:** cluster analysis, provenances, height-diameter models, mixed effects

## Abstract

Chinese fir is the predominant afforestation species in southern China, exhibiting distinct provenances due to long-term climatic adaptation. This study utilized data from four surveys conducted at different ages in a provenance trial forest at Zhangping Wuyi Forest Farm, Fujian Province, to classify Chinese fir provenances using cluster analysis based on growth metrics. The resulting clusters were integrated as random effects into height–diameter models. Model performance was enhanced by incorporating age parameters and validated through five-fold cross-validation. The findings reveal that: (1) the Logistic model best captured the fundamental height–diameter relationship of Chinese fir; (2) the inclusion of provenance-clustering random effects improved model fit and predictive accuracy, with height-based clustering outperforming other methods; (3) the addition of age parameters further refined the base models beyond the clustering effects, and the combination of both approaches achieved the highest precision. Among clustering techniques, height-based clustering surpassed combined height–diameter at breast height (DBH) clustering, while DBH-based clustering was the least effective. The developed models facilitate precise growth predictions for multi-provenance Chinese fir across extensive geographic ranges, offering a theoretical basis for provenance-specific management.

## Simple Summary

Chinese fir (*Cunninghamia lanceolata*), extensively planted across southern China, exhibits marked growth pattern divergence among provenances from different regions, complicating stand-level growth predictions. To address this variation, we analyzed four repeated measurements from successive ages in a provenance trial at Zhangping Wuyi Forest Farm, Fujian Province. Using cluster analysis, provenances were grouped by height, diameter at breast height (DBH), or their combination; these clusters were then incorporated as random effects into height–diameter (H-D) models, with tree age added as a fixed predictor. Height-based clustering yielded the most significant improvements in model fit and predictive accuracy. Further enhancements were achieved when age parameters preceded the integration of height-clustering random effects. The optimized model provides a robust tool for forecasting growth in mixed-provenance Chinese fir stands, enabling informed silvicultural decisions to support sustainable forest management.

## 1. Introduction

Populations of the same tree species often develop distinct ecogeographic types due to prolonged habitat isolation, reproductive barriers, and natural selection, resulting in significant variation in growth traits and environmental adaptability [1]. For instance, Alexandru et al. [2] identified divergent climatic adaptations among Norway spruce (*Picea abies*) provenances, while Jožica et al. [3] demonstrated substantial variation in annual ring width of European beech (*Fagus sylvatica*) provenances. Chinese fir, a vital fast-growing timber species in southern China, is extensively cultivated in plantations for its rapid growth and high-quality wood [4]. Extensive provenance trials have demonstrated that genetic background and habitat heterogeneity collectively contribute to significant genetic variation in physiological traits and growth metrics, such as height, DBH, and volume, across Chinese fir provenances [5,6,7].

Tree height and DBH are fundamental mensurational variables for assessing stand structure and are critical for estimating volume and biomass [8,9,10]. Height–diameter models are indispensable tools in forest resource inventory and management, facilitating predictions of growth dynamics, biomass and carbon estimation, and informed stand optimization [11]. Traditional models, such as Logistic [12], Richards [13], and Weibull [14], describe biological height–diameter relationships through global data fitting. However, these relationships are influenced not only by species-specific traits but also by site conditions [15], competition [16], silvicultural practices [17], and climate change [18], rendering single-growth curves insufficient. Consequently, recent studies have refined these models by incorporating site indices [19,20], competition indices [21], climatic factors [22,23], and silvicultural practices [24]. Mixed-effects models further enhance fit and predictive accuracy by accounting for both population trends and individual variations, achieving greater precision while maintaining parsimony, and have thus gained widespread adoption [25,26].

Current height–diameter models primarily operate at the species level, capturing growth variations across stands and environments but often overlooking provenance-specific influences on growth trajectories at broader scales [27,28,29]. Different provenances may exhibit distinct allometric height–diameter patterns under identical conditions, and ignoring these differences can undermine prediction accuracy. For example, Buford et al. [30] adjusted site index models to account for provenance effects, while Sharma [31] documented significant diameter growth variation in red pine (*Pinus resinosa*) across Canadian regions, highlighting notable provenance differences. Therefore, developing multi-provenance growth models necessitates explicit consideration of provenance divergence and a scientific approach to identifying growth heterogeneity.

Cluster analysis, an unsupervised machine learning technique, provides an objective method to identify distinct growth patterns among provenances based on key traits such as height and DBH, thereby minimizing subjective bias. For instance, previous work has successfully grouped Chinese fir provenances by age class using K-medoids clustering [32] and by similar temperature–humidity responses [33]. These studies underscore the value of cluster analysis in grouping provenances with comparable growth patterns. However, current clustering applications primarily inform provenance selection for afforestation, with limited integration into mechanistic growth models. A deeper investigation into provenance-specific growth patterns holds promise for developing more robust predictive methodologies. Therefore, this study hypothesizes that objectively grouping provenances with similar growth characteristics using cluster analysis can effectively capture this variation. By incorporating these data-driven provenance groups as a random effect in mixed-effects models, we aim to significantly improve the accuracy of height–diameter predictions.

Our primary objective is to evaluate how integrating provenance clustering impacts H-D model predictive performance. Using long-term data from the provenance trial, we developed and tested a clustered mixed-effects model, in which provenance groups identified via cluster analysis were treated as a random effect to account for inherent growth variations. The ultimate goal of this approach is to develop a more accurate, provenance-aware modeling framework, providing theoretical support for precise management and selection of Chinese fir provenances.

## 2. Materials and Methods

### 2.1. Study Area

The study was conducted at Zhangping Wuyi Forest Farm in Fujian Province, China (117°19′–117°38′ E, 25°06′–25°17′ N). The site is located in a subtropical monsoon climate zone characterized by distinct seasons, warm temperatures, and high humidity, with a mean annual temperature ranging from 18 to 20 °C. The mean elevation of the area is 425 m, and soils are predominantly classified as Acrisols (yellow-red soil variant). The forest farm has a standing stock volume of 2.722 million m^3^ and a forest coverage of 91.7%, with plantations of Chinese fir and Masson pine (*Pinus massoniana*) being the dominant forest types.

### 2.2. Data

**Figure 1:**
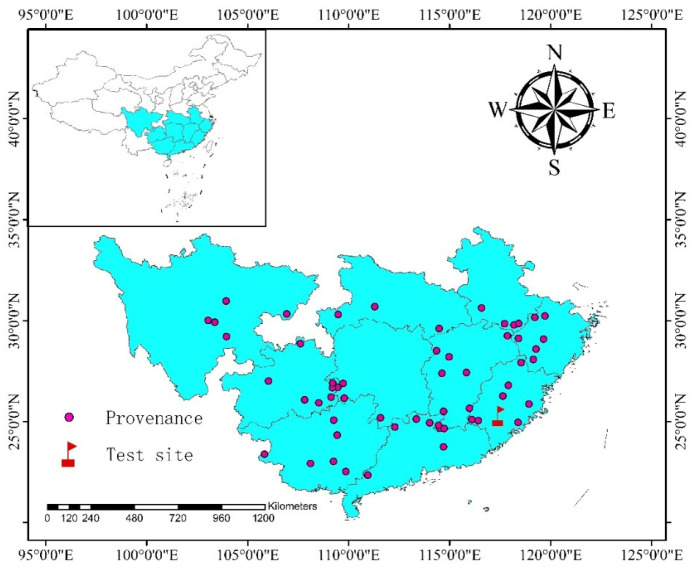
Provenance trial site and geographic origins of the provenances.

**Figure 2:**
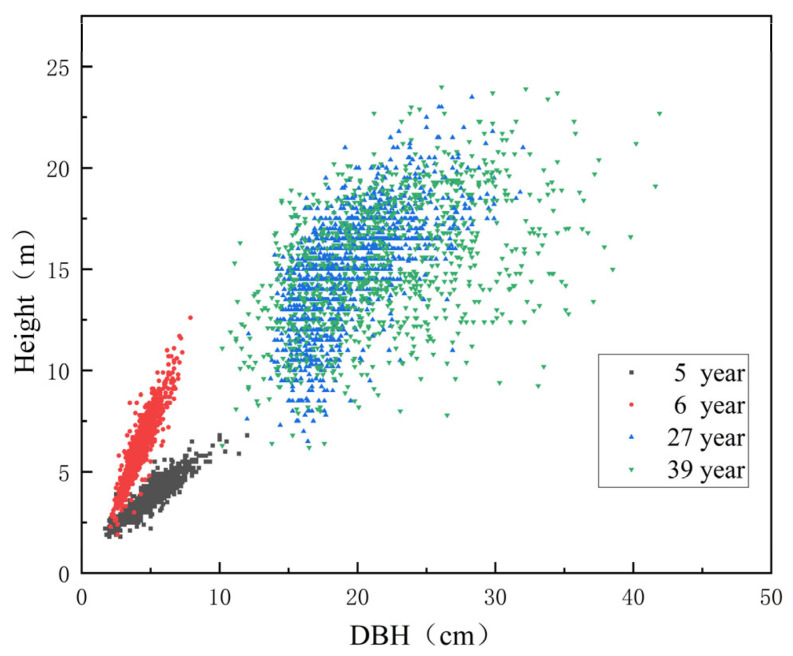
Scatterplot of height–DBH observational data.

Data were obtained from a Chinese fir provenance trial established in 1985 at the Zhangping Wuyi Forest Farm. Figure 1 shows the location of the experimental site and the origin of the provenances. The detailed geographic coordinates and climatic factors for each provenance origin are provided in Appendix A Table A1. Field inventories were conducted in 1989, 1990, 2011, and 2023. For each provenance, ten sample trees of Chinese fir in good condition (i.e., without significant stem curvature) were selected. Following the removal of outliers, a total of 5088 raw datasets encompassing tree height and DBH were acquired (Figure 2).

### 2.3. Cluster Analysis

Growth variations among Chinese fir provenances, resulting from long-term environmental adaptation, exhibit dynamic patterns across developmental stages. This study employed K-means clustering to group provenances, aiming to identify assemblages of provenances exhibiting similar growth phenotypic responses (e.g., DBH, height). Critically, this grouping reflects phenotypic convergence arising from long-term environmental adaptation interacting with genetic backgrounds, rather than constituting a pure genetic provenance classification. For instance, geographically distant provenances may demonstrate comparable growth patterns due to adaptation to analogous environments, consequently being grouped together. Three growth characteristics, namely DBH, height, and their combined metric, were employed to group provenances in this study.

### 2.4. Height–Diameter Model Development

#### 2.4.1. Base Model

Nonlinear models are generally more effective at capturing the biological relationship between tree height and diameter than linear models. Therefore, six biologically meaningful nonlinear models were selected as candidates: Richards, Logistic, Gompertz [34], Curtis [35], Meyer, and Wykoff [36] (Table 1). These six base models have demonstrated superior performance in other foundational model selection studies [37,38,39,40] and represent a range of curve shapes, including sigmoidal and asymptotic, allowing for a comprehensive evaluation of the underlying H-D relationship. The best-performing base model was selected based on a comprehensive evaluation of their fit statistics.

#### 2.4.2. Mixed-Effects Model

A nonlinear mixed-effects modeling approach was employed to account for the hierarchical structure of the data (i.e., multiple trees nested within each provenance cluster). This approach allows us to model both the overall population average trend (fixed effects) and the specific deviations of each provenance cluster from that trend (random effects), thereby accommodating the inherent correlation among measurements within the same cluster and improving model accuracy [25]. The model structure is expressed as

(1) $$H_{ij}=f\left(\beta ,\mu_{i},D_{ij}\right)+\varepsilon_{ij}$$

(2) $$\mu_{i}\sim N(0,G)\;\;\varepsilon_{ij}\sim N(0,R_{ij})$$

where $H_{ij}$ and $D_{ij}$ are the height and DBH, respectively; the *j*-th sample tree is the *i*-th provenance cluster; $f\left(\beta ,\mu_{i},D_{ij}\right)$ is the nonlinear function defined by the selected base model; ***β*** is the vector of fixed-effects parameters; $\mu_{i}$ is the vector of random effects for the *i*-th provenance cluster; G is the variance–covariance matrix for the random effects; $\varepsilon_{ij}$ is the within-group random error term; $R_{ij}$ is the variance–covariance matrix for the within-group error. The variance–covariance matrix is used to describe the variances and pairwise covariances of multiple random variables. It quantifies both the magnitude of differences introduced by different provenance clusters (variance) and whether these differences are correlated across various model parameters (covariance).

To determine the optimal mixed-effects model, random effects were systematically incorporated into every parameter of the selected base model, both individually and in combination. The final model structure was chosen by comparing the goodness-of-fit statistics of these different parameter combinations.

### 2.5. Model Evaluation and Validation

Model performance was assessed using two-fold cross-validation. The Akaike Information Criterion (AIC), Bayesian Information Criterion (BIC), and −2 × log-likelihood (−2LogL) were used to evaluate model fit, with lower values indicating a better fit.

The predictive accuracy of the models was evaluated using the fit index (R^2^), mean absolute error (MAE), mean absolute percentage error (MAPE), and root mean square error (RMSE). Higher R^2^ values and lower values for MAE, MAPE, and RMSE indicate superior model precision and predictive performance. The formulas are as follows:

(3) AIC=2p−2ln(L)

(4) $$BIC=-2\ln\left(L\right)+pln(n)$$

(5) $$2LogL=-2\ln\left(L\right)$$

(6) $$MAE=\frac{\sum_{i=1}^{n}\left|{\hat{H}}_{i}-H_{i}\right|}{n}$$

(7) $$MAPE=\frac{100\%\sum_{i=1}^{n}\left|\frac{{\hat{H}}_{i}-H_{i}}{H_{i}}\right|}{n}$$

(8) $$RMSE=\sqrt{\frac{\sum_{i=1}^{n}\left|{\hat{H}}_{i}-H_{i}\right|}{n}}$$

(9) $$R^{2}=1-\frac{\sum_{i=1}^{n}{\left(H_{i}-{\hat{H}}_{i}\right)}^{2}}{\sum_{i=1}^{n}{\left(H_{i}-{\bar{H}}_{i}\right)}^{2}}$$

where *p* is the number of model parameters, *L* is the maximum value of the likelihood function, *n* is the total number of sample trees, and ${\hat{H}}_{i}$, $H_{i}$, and $\bar{H}$ are the observed, predicted, and mean observed heights for the *i*-th tree, respectively.

### 2.6. Methodology Flowchart

**Figure 3:**
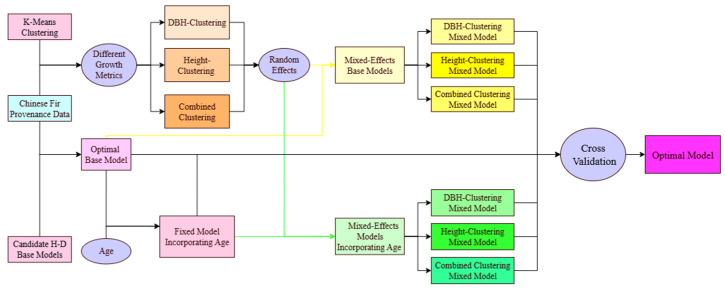
A schematic flowchart of the methodological workflow employed in this study.

A methodological flowchart was created to enhance understanding of the study design (Figure 3).

### 2.7. Statistical Software

All statistical analyses were conducted using R software (version 4.3.2). The K-means clustering was performed using the cluster package (version 2.1.4). The nonlinear mixed-effects models were fitted using the nlme package (version 3.1.162). Figures were generated using the ggplot2 package (version 3.4.4).

## 3. Results

### 3.1. Cluster Analysis Outcomes

K-means clustering was performed on the provenance data using tree height, DBH, and a combined height–DBH metric as the classification variables. For all three approaches, the elbow method consistently identified an optimal cluster number of 3. Consequently, the provenances were classified into three distinct groups for each clustering scheme.

### 3.2. Base Model Selection

Six nonlinear H-D models (Logistic, Richards, Gompertz, Curtis, Meyer, and Wykoff) were evaluated as potential base models. All estimated parameters were statistically significant (*p* < 0.001). The Logistic model yielded the lowest values for all fit statistics (AIC = 1616.889, BIC = 1633.642, and −2LL = 1608.889), as shown in Table 2. Therefore, it was selected as the base model for subsequent mixed-effects modeling of Chinese fir.

### 3.3. Mixed-Effects Models with Different Clustering Approaches

Random effects derived from the three clustering approaches (DBH-based, height-based, and combined) were sequentially incorporated into the Logistic base model. When a single random parameter was added, the greatest model improvement (i.e., lowest fit metrics) was observed with random effects on parameter *c* for height-based and combined clustering, and on parameter *b* for DBH-based clustering. When two random parameters were added, all fit metrics improved further. The optimal two-parameter combination was *a* and *b* for height-based and combined clustering, and *b* and *c* for DBH-based clustering. Models with three random parameters failed to converge.

As shown in Table 3, all mixed-effects models outperformed the base model (M1). Among the clustering methods, height-based clustering (M3) yielded the most substantial reduction in fit statistics, while DBH-based clustering (M2) resulted in the least improvement.

### 3.4. Mixed-Effects Models Incorporating Age and Clustering

The growth pattern of Chinese fir changes with age, exhibiting distinct growth patterns at different ages, and its classification category may shift accordingly. To develop a dynamic H-D model that accounts for these age-dependent relationships while also incorporating provenance-level variation, an age parameter was first incorporated into the base model to create a fixed-effects model (M5) accounting for age-dependent height–diameter relationships. This addition reduced all fit metrics by an average of 18.6% compared to the base model (M1). Subsequently, mixed effects from the three clustering approaches were introduced into M5 by adding random effects (Table 4).

The inclusion of clustering-based random effects further improved model performance, maintaining a consistent hierarchy: height-based clustering provided the greatest enhancement, followed by combined clustering, with DBH-based clustering yielding the smallest improvement. Specifically, the model incorporating both age and height-based clustering (M7) achieved the most substantial improvement, with an average reduction of 60.6% in fit metrics relative to the original base model (M1).

### 3.5. Model Validation

The validation metrics for all developed height–diameter models are presented in Table 5. Model M7, which integrates both age and height-based mixed effects, was identified as the optimal model, ranking first across all validation indices. Models that included the age parameter (M5–M8) consistently outperformed those without it. Furthermore, all mixed-effects models demonstrated superior predictive accuracy compared to their fixed-effects counterparts, with height-based clustering models (M3 and M7) showing the highest accuracy. Table 6 lists the final parameter estimates for the optimal mixed-effects model (M7) fitted to the full dataset.

### 3.6. Model Performance

**Figure 4:**
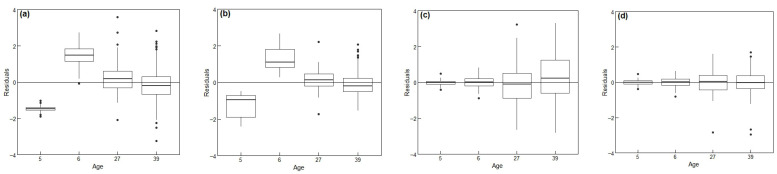
Comparison of residual distributions by tree age for different models. (**a**) The base model (M1), (**b**) the height-clustering mixed model (M3), (**c**) the age-parameterized model (M5), (**d**) the optimal model incorporating age and height-clustering (M7). M7 exhibits the narrowest residual distribution and a stable median, indicating the highest predictive accuracy and reliability, particularly for mature trees. The boxplot elements are defined as follows: central line (median), box boundaries (quartiles), whiskers (1.5 times the interquartile range), and points (outliers).

Figure 4 illustrates the prediction residuals by each age for four key models: the base model (M1), the height-clustering mixed model (M3), the base model incorporating age (M5), and the final model incorporating age and height-clustering (M7). Compared to the M1 (Figure 4a), all other models exhibited residual medians closer to zero. The inclusion of height-based clustering effects (Figure 4b) significantly reduced the dispersion of residuals. Both models incorporating age (Figure 4c,d) displayed stable residual medians near zero across all ages, indicating improved symmetry and reliability. While M7 demonstrated the best overall performance, its prediction errors tended to increase with tree age. A closer comparison reveals that at younger ages (5–6 years), M5 had a smaller residual range than M7; conversely, at later growth stages (27–39 years), M7 showed tighter residual distributions, indicating superior stability for mature trees.

**Figure 5:**
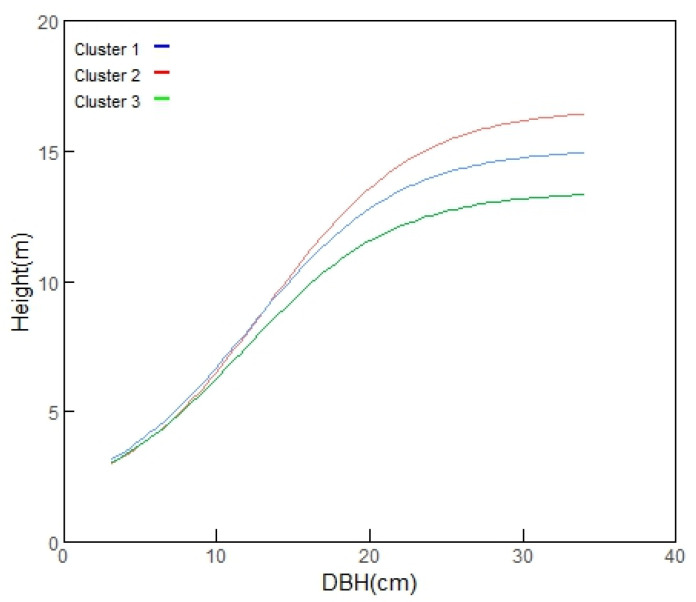
Height growth prediction for the three provenance clusters based on the optimal model (M7).

The height growth curves predicted by the optimal model (M7) for each of the three provenance clusters are displayed in Figure 5. The distinct trajectories for each cluster underscore the importance of provenance classification in accurately modeling the height–diameter relationship in Chinese fir.

## 4. Discussion

This study successfully demonstrates that integrating provenance clustering into mixed-effects models significantly enhances the predictive accuracy of height–diameter relationships in Chinese fir. Our approach addresses a critical challenge in forestry, where genetic divergence among provenances can undermine the reliability of generalized growth models [27,28]. By objectively grouping provenances based on phenotypic growth traits, we developed a model that is more sensitive to these inherent variations. This improved accuracy translates directly into a practical tool for forest managers, enabling them to tailor silvicultural prescriptions (e.g., thinning schedules, rotation age) to specific provenance groups and thereby enhance overall stand productivity and value.

### 4.1. Rationale for Base Model Selection

The six candidate models selected have demonstrated robust performance in extensive previous studies. In this study, the Logistic model exhibited the best results, a finding consistent with some research, such as that by Lumbres et al. [38]. However, the choice of the optimal base model is often species- and site-specific; for instance, Ng’andwe et al. [39] found the Weibull model to be superior, while Patrício et al. [40] reported the Richards model as the top performer. The Logistic model’s success in our context is likely due to its biologically interpretable parameters. Its ability to define distinct growth phases—slow initial increase, rapid acceleration, and asymptotic stabilization—aligns well with the life history of Chinese fir. This biological interpretability facilitates clear comparisons of growth strategies across provenances and is a key contributor to its superior performance.

### 4.2. Superiority of Height-Based Clustering

A key finding of this study was the superior performance of height-based clustering. This underscores the inconsistencies in height–diameter relationships among provenances and highlights the differential efficacy of clustering approaches [41]. The superiority of height-based clustering is likely because maximum height is strongly constrained by site conditions and, in turn, heavily influences H-D allometry [42,43]. As a proxy for site quality, height helps capture provenance-specific adaptations to local edaphic conditions.

Furthermore, height growth is a more direct indicator of a provenance’s adaptation to its native climate. It is primarily governed by apical meristem activity—a process driven by genetic potential and macro-climatic factors such as temperature and photoperiod [23]. In contrast, diameter growth, which is governed by cambial activity, is more susceptible to local stand conditions, including density and resource availability. Consequently, height growth more effectively captures the fundamental climatic adaptations of a provenance, justifying the superior performance of this clustering approach.

### 4.3. Importance of Age for Dynamic Modeling

The inclusion of age as a covariate was crucial for transforming a static H-D relationship into a dynamic model that predicts growth trajectories over time. Our results revealed that the models exhibited lower accuracy at younger ages but superior performance as the stand matured. This discrepancy can be attributed to the higher phenotypic plasticity characteristic of early developmental stages [44]. As the stand ages, genetic control over growth traits strengthen, and heritability increases and stabilizes, as demonstrated by Wu et al. [32] for Chinese fir. This increasing stability of genetically determined growth patterns with age explains why our final model, which accounts for both age and provenance, achieved its highest accuracy in mature trees.

### 4.4. Specification of Random Effects in Mixed Models

Consistent with prior studies [25,26], our clustered mixed-effects models significantly outperformed the base model. While strategies for specifying random effects vary, our analysis revealed that models with two random parameters on the asymptote (a) and a shape parameter (b) performed optimally. This finding is biologically significant: it suggests that the primary differences among the provenance clusters are their maximum growth potential (asymptote) and the rate at which they achieve it (growth curve shape). This aligns with the work of Özçelik et al. [20] and confirms that the optimal allocation of random effects is not merely a statistical choice but can reveal underlying biological differentiation.

### 4.5. Limitations and Future Directions

While our approach is robust, its application is currently limited by the use of data from a single provenance trial. Because provenances exhibit different growth responses when planted in different environments [45], future research should incorporate data from a network of trials across diverse geographic regions to enhance the model’s broader applicability.

Beyond expanding the dataset, future model enhancements could involve incorporating additional explanatory variables. Integrating data on the climate and geography of the provenances’ origins could provide a more comprehensive understanding of the drivers of growth differentiation. Furthermore, including explicit measures of stand competition and dominant height as fixed effects represents a promising avenue for improving predictive accuracy [46,47]. Methodologically, machine learning algorithms offer powerful alternatives for both the initial provenance classification and the final regression modeling [48,49]. Future research could therefore focus on developing hybrid models that integrate machine learning classifiers with a broader set of ecological indicators to advance the next generation of predictive H-D models.

## 5. Conclusions

To address the significant growth divergence among Chinese fir provenances, this study incorporated cluster analysis of key growth traits as a random effect within mixed-effects models. This approach substantially enhanced both model fit and predictive accuracy for height–diameter relationships across these provenances. Notably, height-based clustering yielded optimal improvements. While introducing tree age improved performance beyond the initial cluster-based models, the highest precision was achieved by combining age with cluster-derived random effects. Here again, height-based clustering proved superior. This approach establishes an effective methodology for precise growth modeling in multi-provenance Chinese fir stands and provides a theoretical foundation for efficient, provenance-specific forest management.

## Figures and Tables

**Table 1:** Candidate height–diameter base models for Chinese fir.

Model	Model Equation
Logistic	$H=1.3+a\left(1+be^{-cD}\right)+\varepsilon$
Richards	$H=1.3+{a(1-e^{-bD})}^{c}+\varepsilon$
Gompertz	$H=1.3+e^{-be^{-cD}}$
Curtis	$H=1.3+a\left(\frac{D}{{\left(1+D\right)}^{b}}\right)+\varepsilon$
Meyer	$H=1.3+a\left(1-e^{-bD}\right)+\varepsilon$
Wykoff	$H=1.3+e^{\left(a+b/(D+1)\right)}+\varepsilon$

Note: H represents tree height; D represents diameter at breast height (DBH); a, b, and c are model parameters; ε denotes the error term.

**Table 2:** Fitting results of the six candidate base models.

Model	Parameter Estimation			Fitting Statistic		
	*a*	*b*	*c*	AIC	BIC	−2LogL
Logistic	15.9630 ***	8.3112 ***	0.1979 ***	1616.889	1633.642	1608.889
Richards	18.4873 ***	0.0810 ***	1.3807 ***	1679.122	1695.875	1671.122
Gompertz	17.1237 ***	2.7269 ***	0.1250 ***	1644.014	1660.767	1636.014
Curtis	21.1884 ***	9.0065 ***		1734.280	1746.844	1728.280
Meyer	26.8212 ***	0.0348 ***		1705.752	1718.317	1699.752
Wykoff	3.0956 ***	−10.1254 ***		1718.197	1730.762	1712.197

Note: *** indicates statistical significance at *p* < 0.001.

**Table 3:** Optimal mixed-effects model for different clustering approaches.

No.	Model	Random Parameters	Fixed Parameter			Fitting Statistic		
			*a*	*b*	*c*	AIC	BIC	−2LogL
M1	Base model	None	15.9630	8.3112	0.1979	1616.889	1633.642	1608.889
M2	DBH-clustering mixed model	*b*, *c*	15.8490	8.5890	0.2040	1605.670	1634.987	1591.670
M3	Height-clustering mixed model	*a*, *b*	14.6866	9.9388	0.2554	1490.429	1519.747	1476.429
M4	Combined clustering mixed model	*a*, *b*	15.1953	9.2321	0.2242	1568.430	1597.748	1554.430

**Table 4:** Model fitting with age and clustering-based mixed effects.

No.	Model	Fixed Parameter				Fitting Statistic		
		*a*	*b*	*c*	*c*_0_	AIC	BIC	−2LogL
M5	Incorporating age	13.7433	17.3032	−1.0867	0.2700	1315.050	1335.992	1305.050
M6	Incorporating age and DBH-clustering	13.8381	15.5125	−1.0694	0.2618	1107.400	1140.906	1091.400
M7	Incorporating age and height-clustering	13.7759	12.7433	−1.0737	0.2557	643.648	677.154	627.648
M8	Incorporating age and combined clustering	13.6529	17.3881	−1.1169	0.2760	818.134	851.640	802.134

**Table 5:** Cross-validation results for the different models.

No.	Model	Validation Criterion				Ranking
		RMSE	MAE	MAPE/%	R^2^	
M1	Base model	1.2654	1.0833	17.8629	0.9404	8
M2	DBH-clustering mixed model	1.2426	1.0531	17.1787	0.9425	7
M3	Height-clustering mixed model	1.0936	0.8696	15.4075	0.9555	5
M4	Combined clustering mixed model	1.1996	0.9807	15.7297	0.9464	6
M5	Base model incorporating age	0.9246	0.5852	5.0478	0.9682	4
M6	Incorporating age and DBH-clustering	0.7403	0.4708	4.2767	0.9796	3
M7	Incorporating age and height-clustering	0.4582	0.3145	3.2707	0.9922	1
M8	Incorporating age and combined clustering	0.5510	0.3683	3.5648	0.9887	2

**Table 6:** Parameter estimates of the optimal mixed-effects model.

Parameter	Estimated Value
a	13.7760
b	12.7461
c	−1.0737
$c_{0}$	0.2557
Random effect variance–covariance structure	$\left[\begin{array}{cc} 1.7007 & 2.0700\\ 2.0700 & 2.7865 \end{array}\right]$

## Appendix A

**Table A1:** Geographic coordinates and environmental variables for the provenance origins of Chinese fir.

Province	Provenance Origin	Number of Provenances	Longitude (°N)	Latitude (°E)	Mean Annual Temperature (°C)	Mean Annual Precipitation (mm)
Anhui	Qimen	4	29.85	117.72	15.6	1781
	Qianshan	2	30.63	116.58	16.3	1336
	Xiuning	8	29.79	118.19	17.0	1750
Fujian	Sanming	7	26.27	117.63	19.2	1700
	Pucheng	4	27.92	118.54	13.7	512
	Yangkou	3	26.80	117.91	18.5	1880
Guangdong	Liannan	4	24.73	112.29	19.5	1670
	Xinyi	3	22.35	110.95	22.4	2012
Guangxi	Liuzhou	6	24.33	109.43	20.5	1500
	Liuwanshan	1	22.53	109.86	21.5	1900
	Naping	2	22.93	108.11	18.0	1350
	Zhenglong	2	23.03	109.25	21.5	1477
Guizhou	Jinping	4	26.68	109.20	16.7	1280
	Rongjiang	4	25.93	108.52	18.1	1190
Hubei	Enshi	4	30.3	109.48	16.3	1500
	Tongshan	3	29.61	114.48	17.0	1450
	Yichang	2	30.69	111.29	17.0	1175
Hunan	Huaihua	8	26.69	109.46	16.8	1400
Jiangxi	Ganzhou	2	25.51	114.70	18.9	1500
	Quannan	2	24.82	114.45	17.5	701
	Ruijin	1	25.66	115.98	19.0	1652
	Yichun	8	28.21	114.97	17.6	1642
Sichuan	Hongya	2	29.92	103.37	16.6	1370
	Jianwei	4	29.21	103.95	17.5	1146
	Guxian	4	30.97	103.93	16.1	1200
Zhejiang	Linan	7	30.23	119.72	17.9	1630
	Longquan	8	28.07	119.14	17.8	1650

## References

[1] Rehfeldt G.E., Leites L.P., Clair J.B.S., Jaquish B.C., Sáenz-Romero C., López-Upton J., Joyce D.G. (2014). Comparative Genetic Responses to Climate in the Varieties of Pinus ponderosa and Pseudotsuga menziesii: Clines in Growth Potential. For. Ecol. Manag..

[2] Alexandru A.M., Mihai G., Stoica E., Curtu A.L. (2024). Tree Resilience Indices of Norway Spruce Provenances Tested in Long-Term Common Garden Experiments in the Romanian Carpathians. Plants.

[3] Gričar J., Arnič D., Krajnc L., Peter P., Božič G., Westergren M., Mátyás C., Kraigher H. (2024). Different Patterns of Inter-Annual Variability in Mean Vessel Area and Tree-Ring Widths of Beech from Provenance Trials in Slovenia and Hungary. Trees.

[4] Yu X. (1997). Cunninghamia lanceolata Cultivation.

[5] Gong X., Wan Z., Jin P., Jin S., Li X. (2025). Drought-Driven Divergence in Photosynthetic Performance Between Two *Cunninghamia anceolata* Provenances: Insights from Gas Exchange and Chlorophyll Fluorescence Dynamics. Plants.

[6] Wu P., Tigabu M., Ma X., Odén P.C., He Y., Yu X., He Z. (2011). Variations in Biomass, Nutrient Contents and Nutrient use Efficiency Among Chinese Fir Provenances. Silvae Genet..

[7] Xu T., Niu X., Wang B. (2025). Provenance Differences and Factors Influencing Transpiration of *Cunninghamia lanceolata* in a Common Garden Experiment. Front. Plant Sci..

[8] Dorado F.C., Diéguez-Aranda U., Anta M.B., RodríguezM S., Gadow K. (2006). A Generalized Height-Diameter Model Including Random Components for *Radiata pine* Plantations in Northwestern Spain. For. Ecol. Manag..

[9] Gómez-García E., Diéguez-Aranda U., Castedo-Dorado F., Crecente-Campo F. (2014). A Comparison of Model Forms for the Development of Height-Diameter Relationships in Even-Aged Stands. For. Sci..

[10] Rupšys P. (2015). Height-Diameter Models with Stochastic Differential Equations and Mixed-Effects Parameters. J. For. Res..

[11] Baia A.L.P., Nascimento H.E.M., Guedes M., Hilário R., Toledo J.J. (2025). Tree Height-Diameter Allometry and Implications for Biomass Estimates in Northeastern Amazonian Forests. PeerJ.

[12] Pearl R., Reed L.J. (1920). On the Rate of Growth of the Population of the United States since 1790 and its Mathematical Representation. Proc. Natl. Acad. Sci. USA.

[13] Richards F.J. (1959). A Flexible Growth Function for Empirical Use. J. Exp. Bot..

[14] Weibull W. (1951). A Statistical Distribution Function of Wide Applicability. J. Appl. Mech..

[15] Kuehne C., Maleki K., Merlin M., Granhus A. (2025). Interactive Effects of Species Composition, Site Quality, and Drought on Growth Dynamics of Norway Spruce and *Scots pine* Stands in Norway. For. Ecol. Manag..

[16] Wang J., Wang Y., Zhang Z., Wang W., Jiang L. (2023). Enhanced Awareness of Height-Diameter Allometry in Response to Climate, Soil, and Competition in Secondary Forests. For. Ecol. Manag..

[17] Pukkala T., Lähde E., Laiho O. (2010). Optimizing the Structure and Management of Uneven-Sized Stands of Finland. For. Int. J. For. Res..

[18] Wang X., Fang J., Tang Z., Zhu B. (2006). Climatic Control of Primary Forest Structure and DBH-Height Allometry in Northeast China. For. Ecol. Manag..

[19] Ismail M.J., Poudel T.R., Ali A., Dong L. (2025). Incorporating Stand Parameters in Nonlinear Height-Diameter Mixed-Effects Model for Uneven-Aged *Larix gmelinii* Forests. Front. For. Glob. Change.

[20] Özçelika R., Cao Q.V., Trincadoc G., Göçerd N. (2018). Predicting Tree Height from Tree Diameter and Dominant Height using Mixed-Effects and Quantile Regression Models for Two Species in Turkey. For. Ecol. Manag..

[21] Temesgen H., Gadow K. (2004). Generalized Height-Diameter Models-an Application for Major Tree Species in Complex Stands of Interior British Columbia. Eur. J. For. Res..

[22] Tian D., Jiang L., Shahzad M.K., He P., Wang J., Yan Y. (2022). Climate-Sensitive Tree Height-Diameter Models for Mixed Forests in Northeastern China. Agric. For. Meteorol..

[23] Zhang X., Chhin S., Fu L., Lu L., Duan A., Zhang J. (2019). Climate-Sensitive Tree Height-Diameter Allometry for Chinese Fir in Southern China. For. Int. J. For. Res..

[24] Hao X., Mu C., Cui Y., Ji W., Xu W., Zhao H. (2024). Prediction of Liberation Cutting Intensity Effect on the Growth of Korean pine in Secondary Forest Based on Double Dummy Variable Model. J. Appl. Ecol..

[25] Ciceu A., Leca S., Badea O., Mehtätalo L. (2025). Nonlinear Multilevel Seemingly Unrelated Height-Diameter and Crown Length Mixed-Effects Models for the Southern Transylvanian Forests, Romania. For. Ecosyst..

[26] Sağlam F., Sakici O.E. (2024). Ecoregional Height-Diameter Models for Scots pine in Turkiye. J. For. Res..

[27] Huang Y., Deng X., Zhao Z., Xiang W., Yan W., Ouyang S., Lei P. (2019). Monthly Radial Growth Model of Chinese Fir (*Cunninghamia lanceolata* (Lamb.) Hook.), and the Relationships between Radial Increment and Climate Factors. Forests.

[28] Lu L., Chhin S., Zhang J., Zhang X. (2021). Modelling Tree Height-Diameter Allometry of Chinese Fir in Relation to Stand and Climate Variables through Bayesian Model Averaging Approach. Silva Fenn..

[29] Zhang B., Sajjad S., Chen K., Zhou L., Zhang Y., Yong K.K., Sun Y. (2020). Predicting Tree Height-Diameter Relationship from Relative Competition Levels Using Quantile Regression Models for Chinese Fir (*Cunninghamia lanceolata*) in Fujian Province, China. Forests.

[30] Buford M.A., Burkhart H.E. (1987). Genetic Improvement Effects on Growth and Yield of Loblolly Pine Plantations. For. Sci..

[31] Sharma M. (2021). Modelling Climate Effects on Diameter Growth of *Red pine* Trees in Boreal Ontario, Canada. Trees For. People.

[32] Wu H., Lei J., Li X., Wang H., Duan A., Zhang J. (2021). Aggregation Distributions Across Stand Age in Provenances of *Cunninghamia lanceolata* (Lamb.) Hook. For. Ecol. Manag..

[33] Wang H., Zhu A., Duan A., Wu H., Zhang J. (2022). Responses to Subtropical Climate in Radial Growth and Wood Density of Chinese Fir Provenances, Southern China. For. Ecol. Manag..

[34] Gompertz B. (1825). XXIV. On the Nature of The Function Expressive of The Law of Human Mortality, and on a New Mode of Determining the Value of Life Contingencies. In a letter to Francis Baily, Esq. F. R. S. &c. Philos. Trans. R. Soc. Lond..

[35] Curtis R.O. (1967). Height-Diameter and Height-Diameter-Age Equations for Second-Growth Douglas-Fir. For. Sci..

[36] Robinson A.P., Wykoff W.R. (2004). Imputing Missing Height Measures using a Mixed-Effects Modeling Strategy. Can. J. For. Res..

[37] Mensah S., Pienaar O.L., Kunneke A., Toit B., Seydack A., Uhl E., Pretzsch H., Seifert T. (2018). Height-Diameter Allometry in South Africa’s Indigenous High Forests: Assessing Generic Models Performance and Function Forms. For. Ecol. Manag..

[38] Lumbres R.L.C., Lee Y.J., Calora F.G., Parao M.R. (2013). Model Fitting and Validation of Six Height–DBH Equations for *Pinus kesiya* Royle ex Gordon in Benguet Province, Philippines. For. Sci. Technol..

[39] Ng’andwe P., Chungu D., Yambayamba A.M., Chilambwe A. (2019). Modeling the Height-Diameter Relationship of Planted Pinus kesiya in Zambia. For. Ecol. Manag..

[40] Patrício M.S., Dias C.R.G., Nunes L. (2022). Mixed-effects Generalized Height-Diameter Model: A Tool for Forestry Management of Young Sweet Chestnut Stands. For. Ecol. Manag..

[41] Wu H., Duan A., Zhang J. (2019). Long-Term Growth Variation and Selection of Geographical Provenances of *Cunninghamia lanceolata* (Lamb.) Hook. Forests.

[42] Noordermeer L., Bollandsås O.M., Gobakken T., Næsset E. (2018). Direct and Indirect Site Index Determination for Norway Spruce and Scots pine using Bitemporal Airborne Laser Scanner Data. For. Ecol. Manag..

[43] Sharma M., Parton J. (2007). Height-Diameter Equations for Boreal Tree Species in Ontario using a Mixed-Effects Modeling Approach. For. Ecol. Manag..

[44] Wu H., Duan A., Zhang J. (2019). Growth Variation and Selection Effect of *Cunninghamia lanceolata* Provenances at Different Stand Ages. For. Sci..

[45] Wang H., Duan A., Zhang J. (2025). Intraspecific Responses to Climate Change in *Cunninghamia lanceolata* (Lamb.) Hook.: Local May Not be the Best. For. Ecol. Manag..

[46] Köhler P., Huth A. (1998). The Effects of Tree Species Grouping in Tropical Rainforest Modelling: Simulations with The Individual-Based Model FORMIND. Ecol. Model..

[47] Phillips P.D., Yasman I., Brash T.E., Gardingen P.R. (2022). Grouping Tree Species for Analysis of Forest Data in Kalimantan (Indonesian Borneo). For. Ecol. Manag..

[48] Özçelik R., Diamantopoulou M.J., Crecente-Campo F., Eler U. (2013). Estimating Crimean Juniper Tree Height Using Nonlinear Regression and Artificial Neural Network Models. For. Ecol. Manag..

[49] Ou Y., Quiñónez-Barraza G. (2023). Modeling Height–Diameter Relationship Using Artificial Neural Networks for Durango Pine (*Pinus durangensis* Martínez) Species in Mexico. Forests.

